# Supplementary material for: Phytochemical profiling and antioxidant activity assessment of Bellevalia pseudolongipes via liquid chromatography-high-resolution mass spectrometry
Source: PeerJ. 2024 Sep 13;12:e18046. doi: 10.7717/peerj.18046 (PMC11404456; doi:10.7717/peerj.18046)
Supplement: Supplemental Information 2 [file peerj-12-18046-s002.pdf]

BİNGÖL ÜNİVERSİTESİ MERKEZİ LABORATUVAR
UYGULAMA VEARAŞTIRMA MERKEZİ
/ BINGOL UNIVERSITY CENTRAL LABORATORY APPLICATION
AND RESEARCH CENTER
Selahaddin-i Eyyubi Mah., Aydınlık Cad. No:1, 12000 Bingöl Merkez/Bingöl
((0426) 216 00 12-dahili-5016)
http://bingol.edu.tr
bumlab@bingol.edu.tr
IBAN: TR 2200 0120 0963 5000 0600 0060

LC-MS ORBITRAP (SIVI KROMATOGRAFİSİ KÜTLE SPEKTROMETRESİ) 91 adet'e Kadar Fenolik Bileşikler Analizi Analiz Raporu

Müşteri Bilgileri	
Müşteri adı/ Customer name	İdris YOLDAŞ
Adres/ Address	SİİRT ÜNİVERSİTESİ
Rapor Teslim Adresi/ Report Delivery Address	
Vergi Dairesi No	
Telefon/ Telephone	
e-posta/ email	

Örnek Bilgileri/ Sample Information				
Örnek Kodu ve Tanımı/ Sample Code and Description	2 adet (katı halde/ solid state)<br>A16			
Kayıt No/ Registration number	Örnek Sayısı/ Number of Samples	Kayıt Tarihi/ Date of registration	Analiz Tarihi/ Analysis Date	Rapor Tarihi/ Report Date
İ950	2 adet	27.12.2022	13.02.2023	20.02.2023

Analiz Sonuçları/ Analysis Results

Sıvı Kromatografisi-Yüksek Çözünürlük Kütle Spektrometrisi (LC-HRMS) Cihazı

LC-HRMS analizleri, DIONEX UltiMate 3000 RS pompası, DIONEX UltiMate 3000 RS otomatik numune alıcısı ve DIONEX UltiMate 3000 RS kolon fırını içeren LC sistemi ve ısıtmalı elektrosprey iyonlaşma arayüzüne sahip Exactive Plus Orbitrap (Thermo Fisher Scientific) yüksek çözünürlüklü MS bileşimi kullanılarak gerçekleştirilmiştir. Orbitrap-MS cihazı, bir otomatik şırınga enjektörü (Thermo Fisher Scientific, ABD) kullanılarak pozitif (Pierce™ LTQ Velos ESI Positive Ion Calibration Solution) ve negatif kalibrasyon (Pierce™ Negative Ion Calibration Solution) solüsyonları ile kalibre edilmiştir. Yapılan LC-HRMS analizlerinde LC ve MS kısmı sistem bilgisayarına yüklenmiş olan TraceFinder 3.2 (Thermo Scientific) programı ile eş zamanlı olarak çalıştırılmış, veriler Xcalibur software version 2.1.0.1140 (Thermo Fisher Scientific) programı ile toplanılarak kaydedilmiştir.

Kromatografi ve Yüksek Çözünürlüklü MS Koşulları

Gerçekleştirilen analizler bir Phenomenex® Gemini® 3µm NX-C18 110 Å (100 mm × 2mm) kolon kullanıldı. Kolon fırın sıcaklığı 30 °C olarak çaıştırıldı. Elüsyon gradiyentinde hareketli A fazında

BİNGÖL ÜNİVERSİTESİ MERKEZİ LABORATUVAR
UYGULAMA VEARAŞTIRMA MERKEZİ
/ BINGOL UNIVERSITY CENTRAL LABORATORY APPLICATION
AND RESEARCH CENTER
Selahaddin-i Eyyubi Mah., Aydınlık Cad. No:1, 12000 Bingöl Merkez/Bingöl
((0426) 216 00 12-dahili-5016)
http://bingol.edu.tr
bumlab@bingol.edu.tr
IBAN: TR 2200 0120 0963 5000 0600 0060

Ultrapure su sistemi (GFL 2004/ Human power 1) ile elde edilen ultra safsu da hazırlanmış % 2 (v/v) glasiyel asedik asit, hareketli B fazında %99,9 saflıkta LC-MS derecede metanol (Sigma) kullanılmıştır. Ayırım, numune enjeksiyon hacmi 20,0 µL ve 0,3 mL/dk akış hızında gradiyent elüsyon koşulları Şekil 1'de belirtilen durumlara göre gerçekleştirildi. Analiz süresi toplam 20 dakika olarak ayarlandı.

	Retention [min]	Flow [ml/min]	%B
1	0.000	0.300	0.0
2	0.000	0.300	0.0
3	2.000	0.300	0.0
4	13.000	0.300	98.0
5	15.900	0.300	98.0
6	16.000	0.300	0.0
7	19.000	0.300	0.0

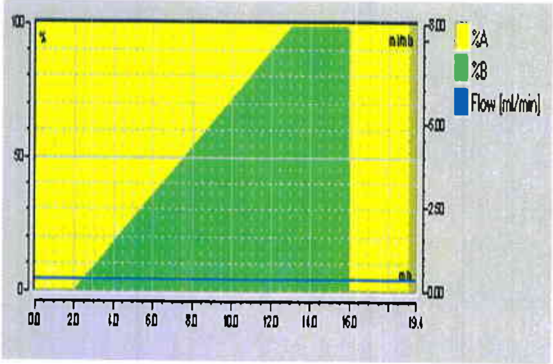

Şekil 1. Elüsyonda takip eden gradiyent koşulları.

Isıtılmış bir elektrosprey iyonlaşma ara yüzü ile donatılmış Orbitrap HRMS hem pozitif (Full MS/AIF) hem de negatif (Full MS/AIF) modda çalıştırıldı. İyonlaştırma ara yüzü kılıf gaz (sheath gas) akış oranı 35; yardımcı gaz (auxiliarygas) akış oranı 7; sprey voltaj 3,5 kV; kapiler sıcaklığı 350 °C; yardımcı gaz (auxiliarygas) sıcaklığı 350 °C; S-lens RF seviyesi 50 olarak ayarlandı. MS tarama aralığı 60-800 m/z; resolution 17500; ACG target 3e6; maximum IT 2 ms; CE (çarpışma enerjisi, collision energy)/ step CE 25 V koşullarında gerçekleştirildi.

Örnekler 10 mg tartılıp üzerine hacimce metanol:su 1:1 karışımından 10 mL ilave edilerek çözüldü. 0.22 µm 25mm çaplı PTFE şırınga filtreden 1,5 mL viale süzülerek analiz yapıdı.

Fenolik Bileşik Profili/ Phenolic Compound Profile	Sonuç/Result	Birim/Unit	METOT/ Method
101-)Benzoic acid	48,755	mg/kg	LC-MS/MS
102-) 4-Hydroxybenzoic acid	8,709	mg/kg	LC-MS/MS
103-) Salicylic acid	N/F	mg/kg	LC-MS/MS
104-)3-hydroxybenzoic acid (3-HBA)	0,186	mg/kg	LC-MS/MS
105-)3-hydroxyphenylacetic acid (3-HPA)	221,558	mg/kg	LC-MS/MS
106-)Syringic acid	14,118	mg/kg	LC-MS/MS
107-)Gallic acid(3,4,5-trihydroxybenzoic acid)	10,499	mg/kg	LC-MS/MS
108-)Protocatechuic acid (3,4-Dihydroxybenzoic acid)	6,962	mg/kg	LC-MS/MS
109-)Protocatechuic acid ethyl ester (Ethyl 3,4-Dihydroxybenzoate)	N/F	mg/kg	LC-MS/MS

110-)3,4-dihydroxybenzaldehyde (Protocatechuic aldehyde)	24,666	mg/kg	LC-MS/MS
111-)2,4-dihydroxybenzoic acid (beta-Resorcylic acid)	N/F	mg/kg	LC-MS/MS
112-)Vanillic acid	35,353	mg/kg	LC-MS/MS
113-)Homovanillic acid((4-Hydroxy-3-methoxyphenylacetic acid)	27,639	mg/kg	LC-MS/MS
114-)Vanillin	29,377	mg/kg	LC-MS/MS
115-)Gentisic acid	N/F	mg/kg	LC-MS/MS
116-)3,4-Dihydroxyphenylacetic acid(DOPAC, Homoprotocatechuic acid)	35,353	mg/kg	LC-MS/MS
117-)trans Cinnamic acid	3331,911	mg/kg	LC-MS/MS
118-)coumaric acid (trans-3-Hydroxycinnamic acid)	2,503	mg/kg	LC-MS/MS
119-)Caffeic acid	259,684	mg/kg	LC-MS/MS
120-)Caffeic acid phenhyl ester (CAPE)	N/F	mg/kg	LC-MS/MS
121-)Ferulic acid	N/F	mg/kg	LC-MS/MS
122-)Sinapic acid	N/F	mg/kg	LC-MS/MS
123-)Chlorogenic acid	N/F	mg/kg	LC-MS/MS
124-)Quinic acid	13,9	mg/kg	LC-MS/MS
127) α-Cyano-4-hydroxycinnamic acid	N/F	mg/kg	LC-MS/MS
128-)Catechin (Cianidanol)-p	N/F	mg/kg	LC-MS/MS
129-)Epigallocatechin	N/F	mg/kg	LC-MS/MS
130-)Epigallocatechin gallate	N/F	mg/kg	LC-MS/MS
131-)Chrysin (5,7-Dihydroxy-2-phenyl-4H-chromen-4-one)	N/F	mg/kg	LC-MS/MS
132-)Apigenin (5,7-Dihydroxy-2-(4-hydroxyphenyl)-4H-chromen-4-one)	N/F	mg/kg	LC-MS/MS
133-)Acacetin (5,7-Dihydroxy-2-(4-methoxyphenyl)-4H-chromen-4-one)	N/F	mg/kg	LC-MS/MS
134-)Rhoifolin (Apigenin 7-O- neohesperidoside)	N/F	mg/kg	LC-MS/MS
135-)Vicenin 2	N/F	mg/kg	LC-MS/MS
136-) Apigenin 7-glucuronide	N/F	mg/kg	LC-MS/MS
137-) Apigenin 7-glucoside	15,696	mg/kg	LC-MS/MS
138-)Genkwanin(4',5-Dihydroxy-7-metthoxyflavone, Apigenin 7-O-methyl ether)	N/F	mg/kg	LC-MS/MS
139-) Apiin (Apigenin-7-(2-O-apiosylglucoside)	79,875	mg/kg	LC-MS/MS
140-) Vitexin (Apigenin 8-C-glucoside)	277,122	mg/kg	LC-MS/MS
141-)Schaftoside	837,932	mg/kg	LC-MS/MS
143-) Rutin hydrate M-OH2	N/F	mg/kg	LC-MS/MS
144-) Luteolin	124,969	mg/kg	LC-MS/MS
145-) Luteolin-7-O-glucuronide (Luteolin-7-O-β-D-glucuronide)	N/F	mg/kg	LC-MS/MS
146-) Diosmetin (Luteolin 4'-methyl ether)	12,85	mg/kg	LC-MS/MS
147-) Orientin	1308,652	mg/kg	LC-MS/MS
148-) Isoorientin	1308,652	mg/kg	LC-MS/MS
149-) Luteoloside (Luteolin 7-glucoside)	78,76	mg/kg	LC-MS/MS
150-) Luteolin 7-rutinoside	32,313	mg/kg	LC-MS/MS
151-) Galangin (3,5,7-Trihydroxy-2-phenyl-4H-chromen-4-one)	N/F	mg/kg	LC-MS/MS
152-) Quercetin	8,241	mg/kg	LC-MS/MS
153-) İsoquercitrin (Quercetin 3-glucoside)	43,656	mg/kg	LC-MS/MS
154-) Narcissin (Narcissoside, Isorhamnetin 3-rutinoside)	N/F	mg/kg	LC-MS/MS
155-) Quercetin 3-rutinoside 7-glucoside	N/F	mg/kg	LC-MS/MS
156-) Isorhamnetin (Quercetin 3'-methyl ether)	N/F	mg/kg	LC-MS/MS
157-) Hyperoside (Quercetin 3-D-galactoside)	43,656	mg/kg	LC-MS/MS
158-) Kaempferol	N/F	mg/kg	LC-MS/MS
159-) Afzelin (Kaempferol 3-rhamnoside)	3,674	mg/kg	LC-MS/MS
160-) Kaempferide	N/F	mg/kg	LC-MS/MS
161-) Kaempferitrin	N/F	mg/kg	LC-MS/MS
162-) Nicotiflorin (Kaempferol 3-rutinoside, Kaempferol 3-O-β –rutinoside)	N/F	mg/kg	LC-MS/MS

Selahaddin-i Eyyubi Mah., Aydınlık Cad. No:1, 12000 Bingöl Merkez/Bingöl
((0426) 216 00 12-dahili-5016)
http://bingol.edu.tr
bumlab@bingol.edu.tr
IBAN: TR 2200 0120 0963 5000 0600 0060

163-) Astragalin (Kaempferol 3-glucoside)	N/F	mg/kg	LC-MS/MS
164-) Tiliroside	N/F	mg/kg	LC-MS/MS
165-) Leucoside (Kaempferol 3-sambubioside)	N/F	mg/kg	LC-MS/MS
167-) Fisetin hydrate	2,304	mg/kg	LC-MS/MS
168-) Naringin	N/F	mg/kg	LC-MS/MS
169-) Naringenin	N/F	mg/kg	LC-MS/MS
170-) Sakuranetin (Naringenin 7-O-methyl ether)	N/F	mg/kg	LC-MS/MS
171-) Narirutin (Narirutinsa, Naringenin rutinoside)	472,752	mg/kg	LC-MS/MS
173-) Hesperidin	0,009	mg/kg	LC-MS/MS
174-) Neohesperidin	0,009	mg/kg	LC-MS/MS
175-) Eriodictyol (3,4,5,7-Tetrahydroxyflavanone)	N/F	mg/kg	LC-MS/MS
176-) Eriocitrin	N/F	mg/kg	LC-MS/MS
177-) Liquiritigenin	N/F	mg/kg	LC-MS/MS
178-) Liquiritin (4',7-Dihydroxyflavanone 4'-glucoside)	N/F	mg/kg	LC-MS/MS
179-) Genistein (5,7-Dihydroxy-3-(4-hydroxyphenyl)-4H-chromen-4-one)	N/F	mg/kg	LC-MS/MS
180-) Daidzin	N/F	mg/kg	LC-MS/MS
181-) Formononetin (Neochanin)	N/F	mg/kg	LC-MS/MS
182-) Kuromanine (Cyanidin 3-glucoside chloride)	N/F	mg/kg	LC-MS/MS
183-) ellagic acid	74,263	mg/kg	LC-MS/MS
184-) Esculin hydrate	1,643	mg/kg	LC-MS/MS
185-) Phloridzin	N/F	mg/kg	LC-MS/MS
186-) Rosmarinic acid	14,426	mg/kg	LC-MS/MS
187-) Glabridin	N/F	mg/kg	LC-MS/MS
188-) Arbutin	85,031	mg/kg	LC-MS/MS
189-) emodin	N/F	mg/kg	LC-MS/MS
190-) Etoposide	N/F	mg/kg	LC-MS/MS
191-) Procyanidin B2	7,652	mg/kg	LC-MS/MS
192-) Doxorubicin Hydrchloride	N/F	mg/kg	LC-MS/MS
193-) ethylgallate	N/F	mg/kg	LC-MS/MS

AÇIKLAMALAR

- Analiz sonuçları yalnızca laboratuvara teslim edilen numuneye aittir. Numunenin alınmasından laboratuvara teslim edilmesine kadar olan süreçten ve numunenin temsil özelliğinden laboratuarımız sorumlu değildir.
- Numune, BÜMLAB tarafından teslim alındığı andan itibaren analiz zamanına kadar uygun koşullarda ve sürede saklanarak analiz sürecine alınmıştır.
- İmzasız ve mühürsüz raporlar geçersizdir.

**Analiz Personeli/ Analysis Staff**	**Kalite Yönetim Sorumlusu/ Quality Management Officer**	**BÜMLAB Müdürü/** APPROVED BY CENTER MANAGER
**Dr. Ögr. Üyesi İnan DURSUN**	**Öğr. Gör. Buket ARICI**	**Doç. Dr. Ferdi AKMAN**
