# Supplementary material for: Phytochemical profiling and antioxidant activity assessment of Bellevalia pseudolongipes via liquid chromatography-high-resolution mass spectrometry
Source: PeerJ. 2024 Sep 13;12:e18046. doi: 10.7717/peerj.18046 (PMC11404456; doi:10.7717/peerj.18046)

High Density Calibration Report

**Lab Name:**	Default Laboratory		
**Instrument:**	Thermo Scientific Instrument	**Method:**	FENOLICQUAN-13_FENILICQUAN
**User:**	Thermo		FENILICQUAN
**Batch:**	FENOLICQUAN-13	**Cali File:**	FENOLICQUAN-13.calx

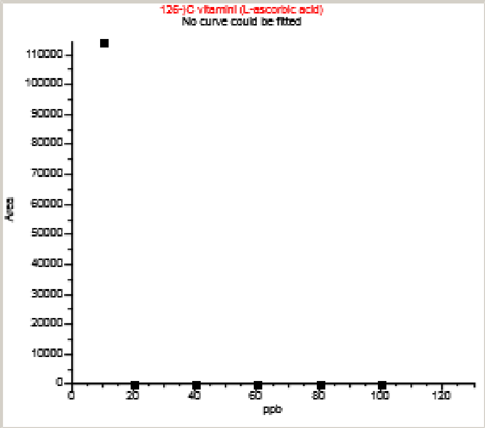

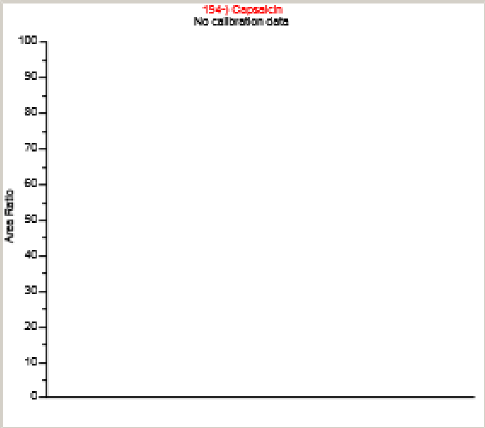

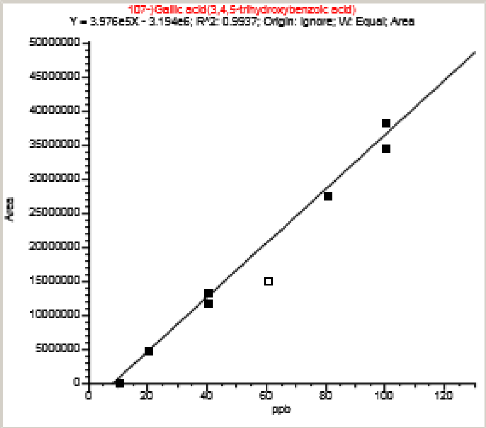

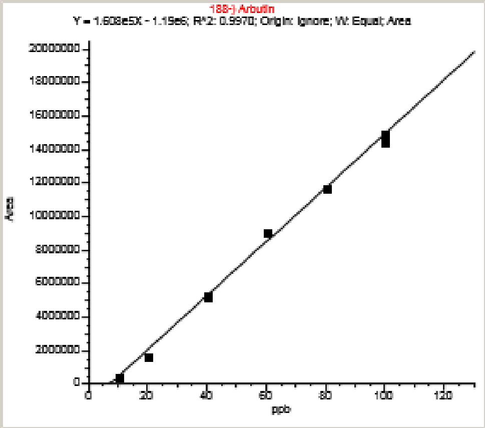

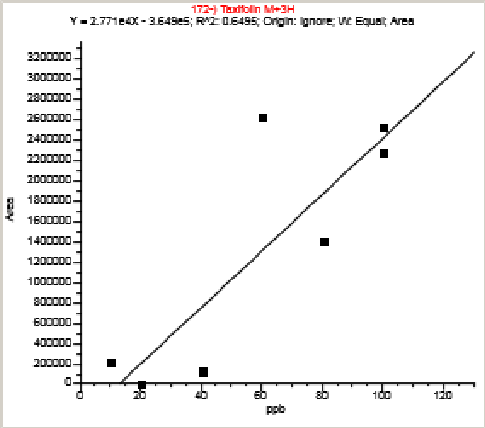

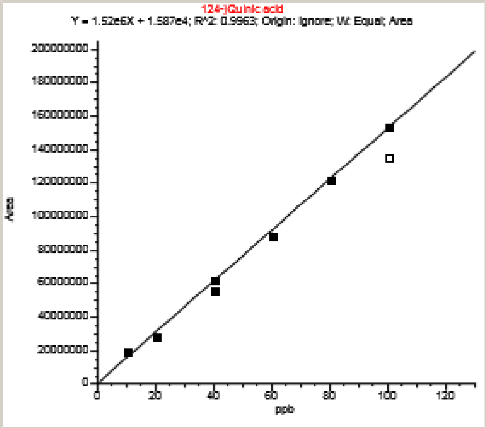

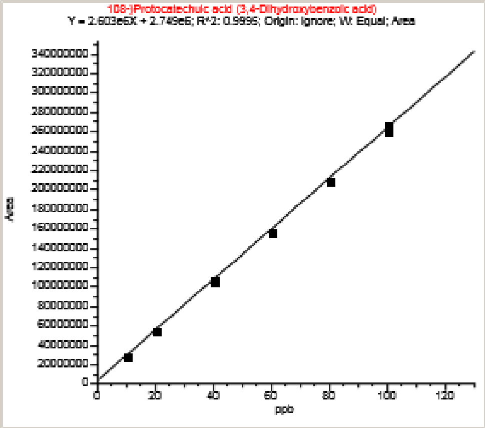

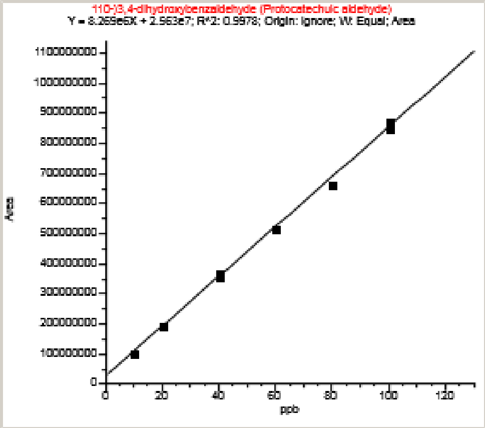

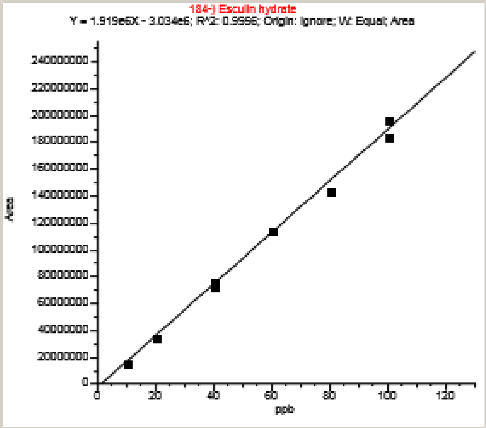

High Density Calibration Report

Lab Name: Default Laboratory
Instrument: Thermo Scientific Instrument
User: Thermo
Batch: FENOLICQUAN-13
Method: FENOLICQUAN-13_FENILICQUAN FENILICQUAN
Cali File: FENOLICQUAN-13.calx

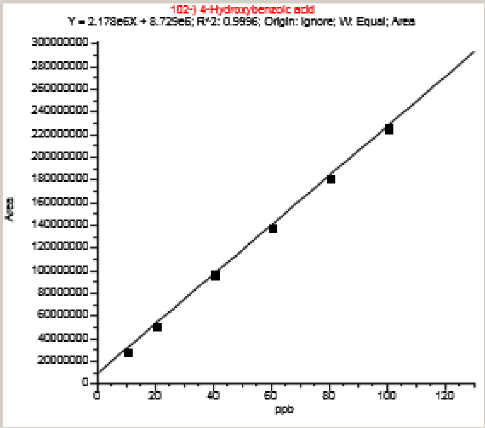

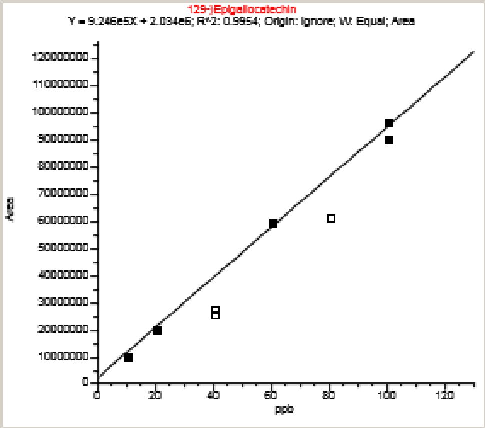

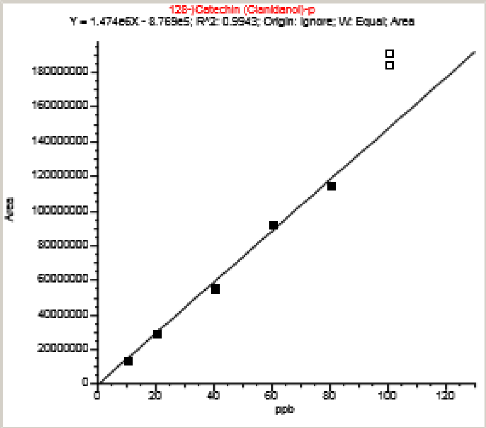

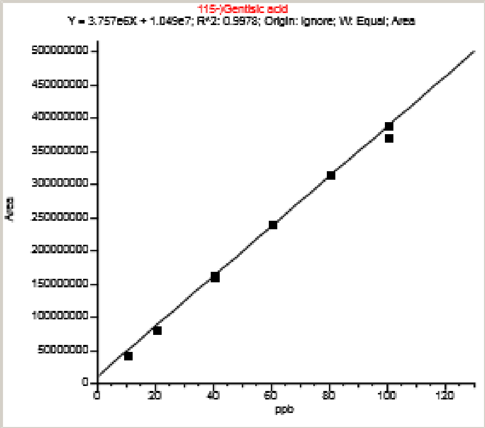

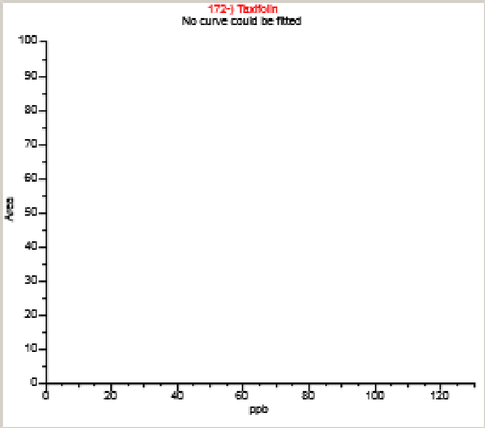

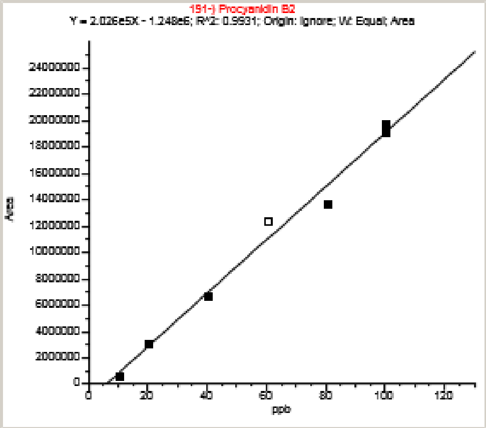

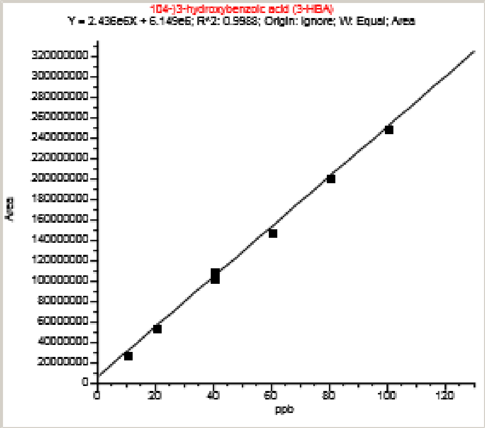

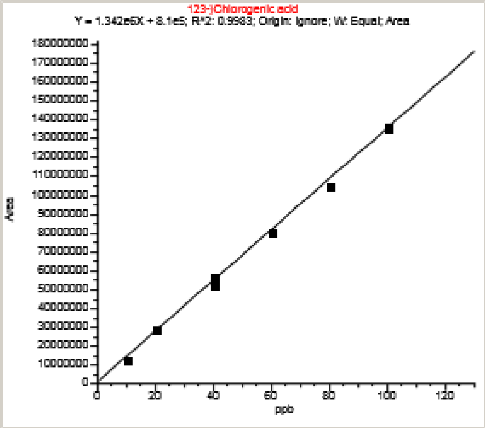

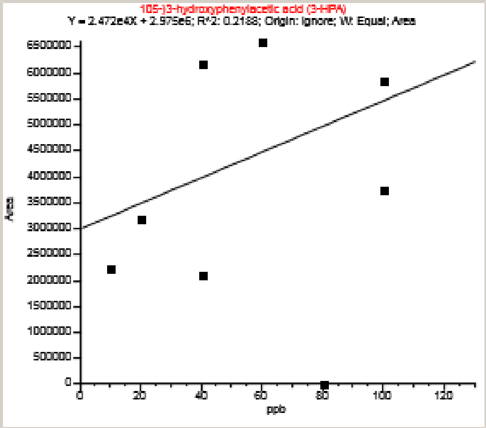

High Density Calibration Report

Lab Name: Default Laboratory
Instrument: Thermo Scientific Instrument
User: Thermo
Batch: FENOLICQUAN-13

Method: FENOLICQUAN-13_FENILICQUAN
FENILICQUAN
Cali File: FENOLICQUAN-13.calx

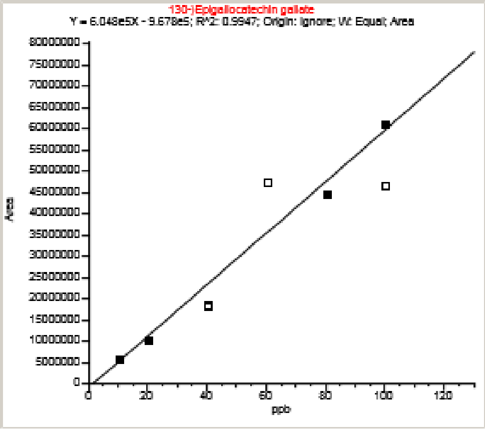

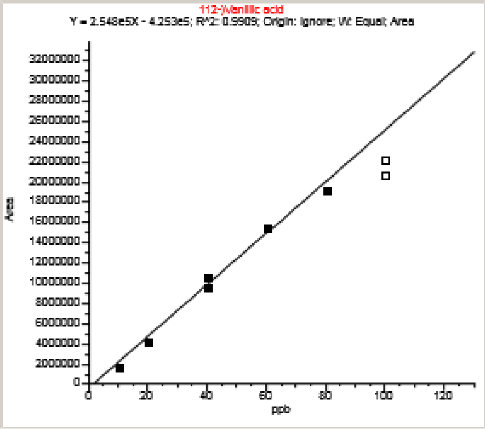

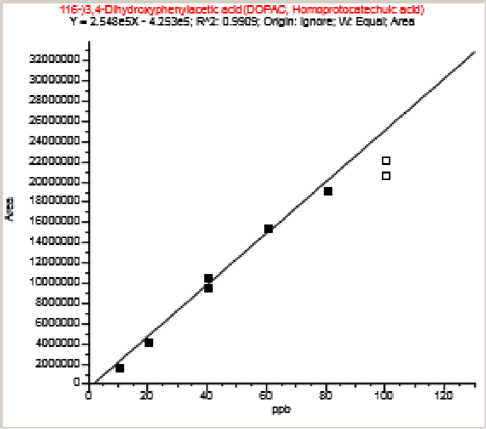

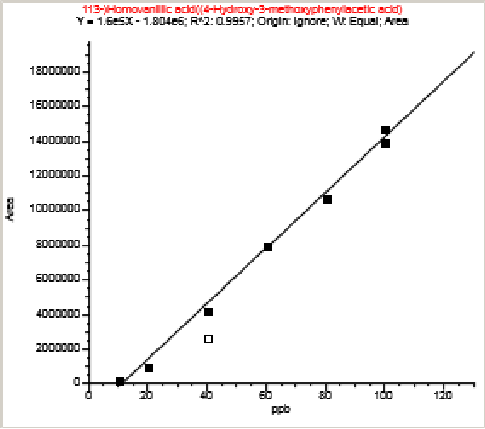

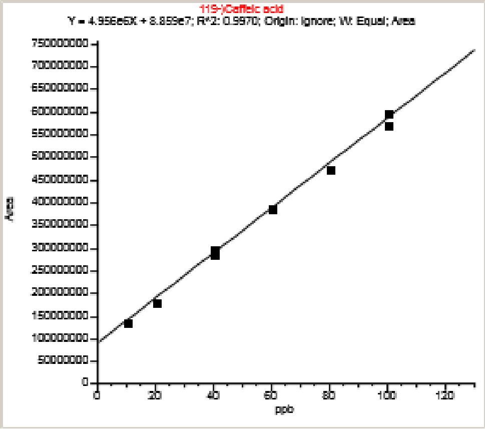

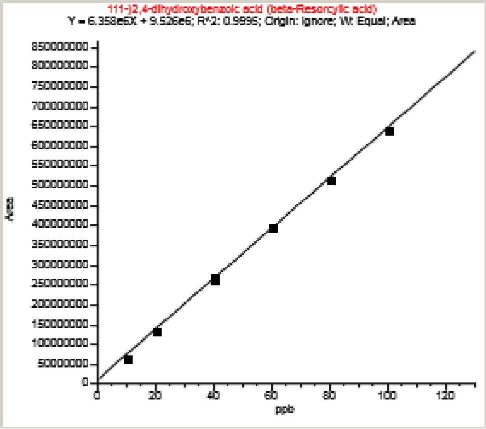

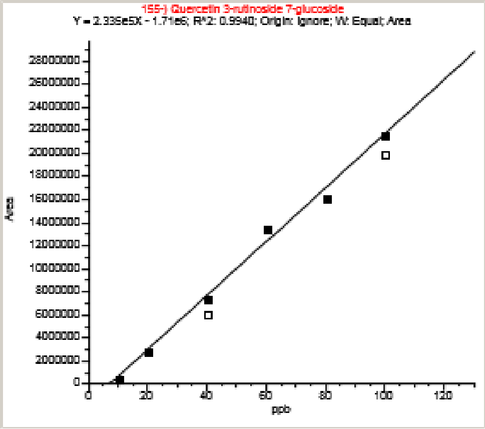

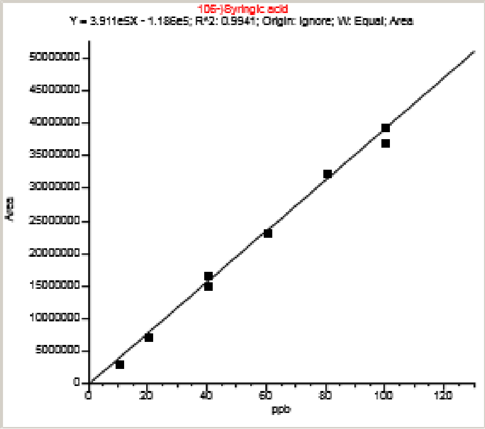

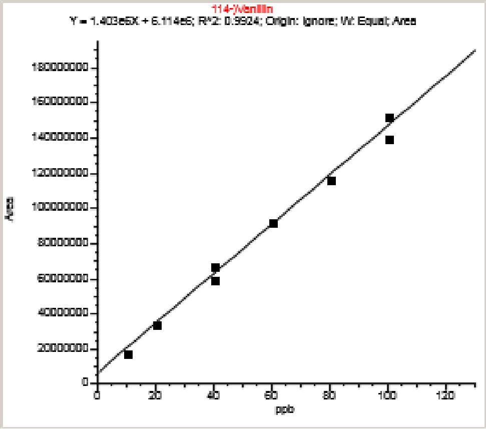

High Density Calibration Report

Lab Name: Default Laboratory
Instrument: Thermo Scientific Instrument
User: Thermo
Batch: FENOLICQUAN-13
Method: FENOLICQUAN-13_FENILICQUAN FENILICQUAN
Cali File: FENOLICQUAN-13.calx

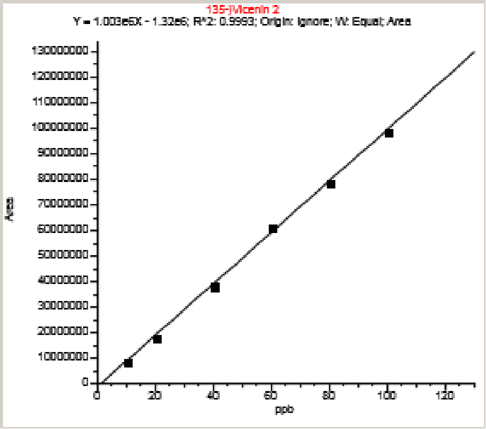

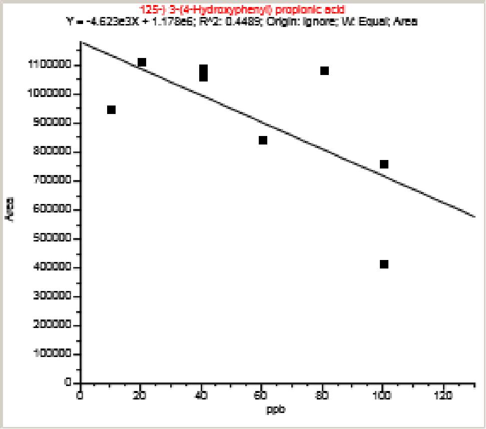

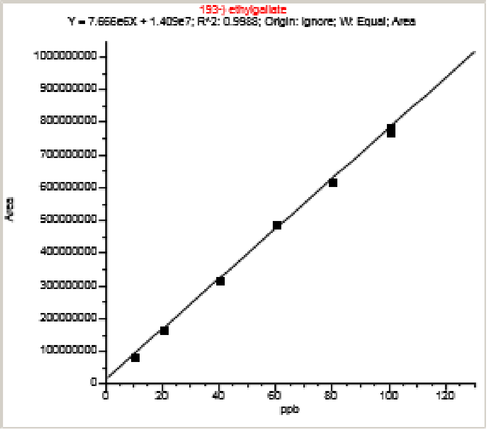

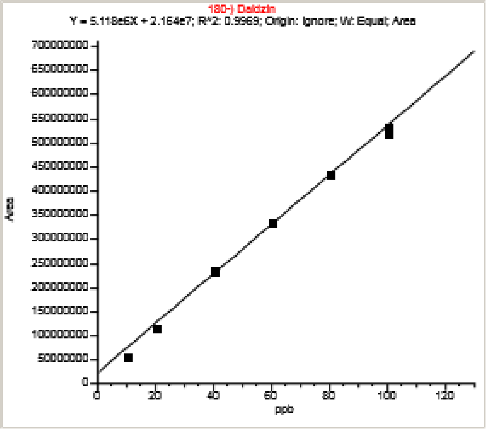

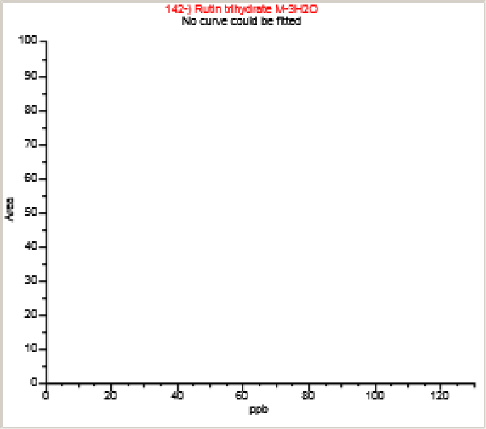

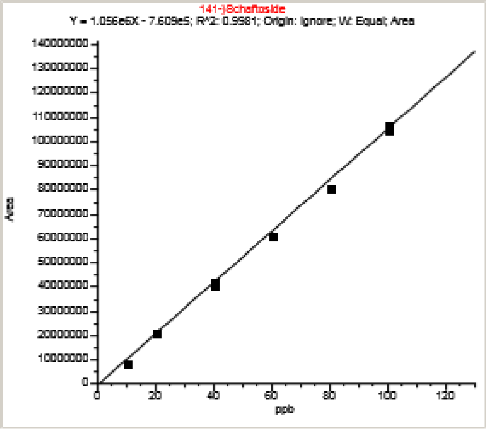

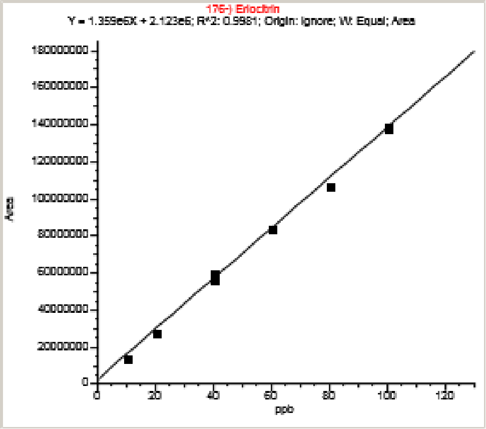

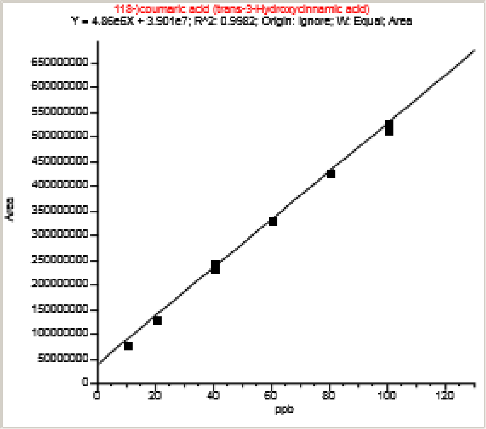

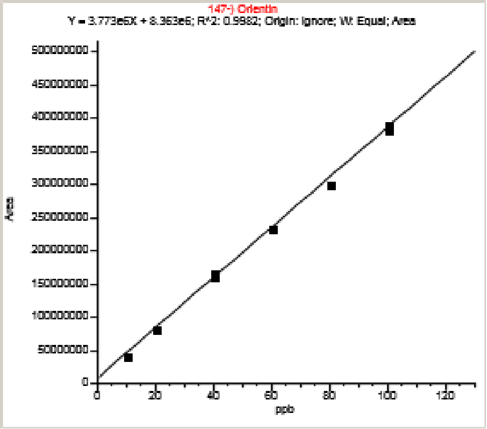

High Density Calibration Report

Lab Name: Default Laboratory
Instrument: Thermo Scientific Instrument
User: Thermo
Batch: FENOLICQUAN-13

Method: FENOLICQUAN-13_FENILICQUAN
FENILICQUAN
Cali File: FENOLICQUAN-13.calx

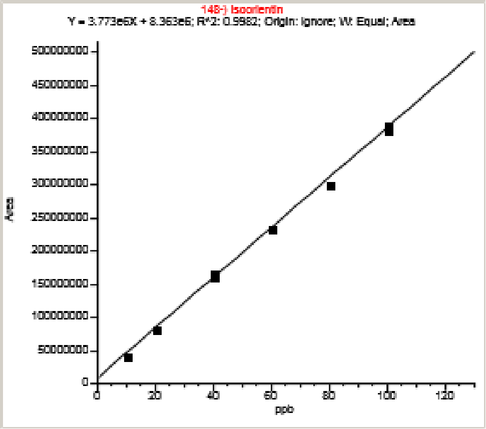

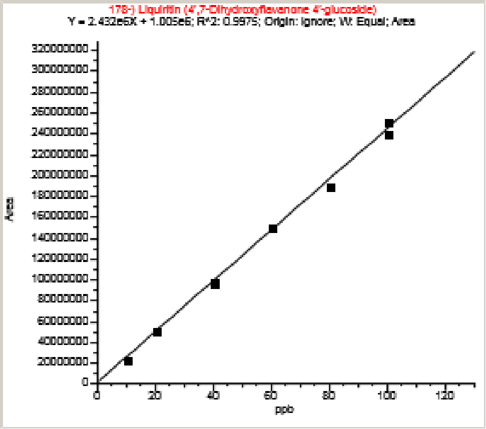

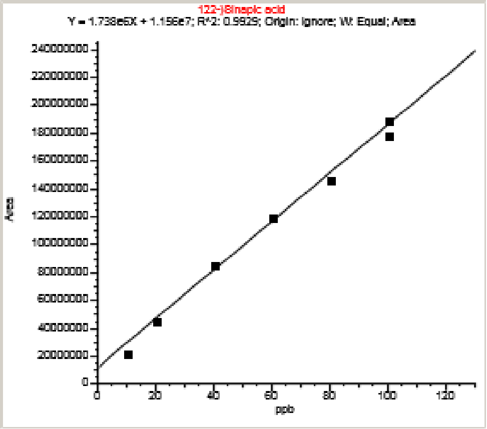

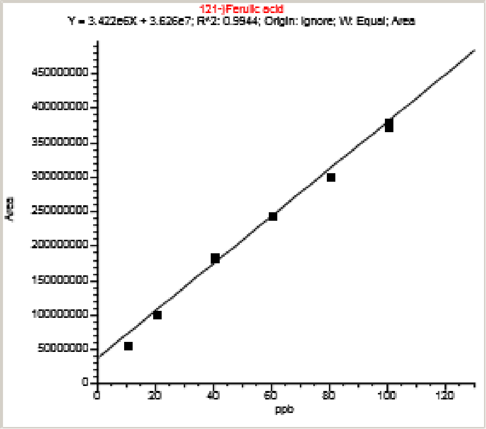

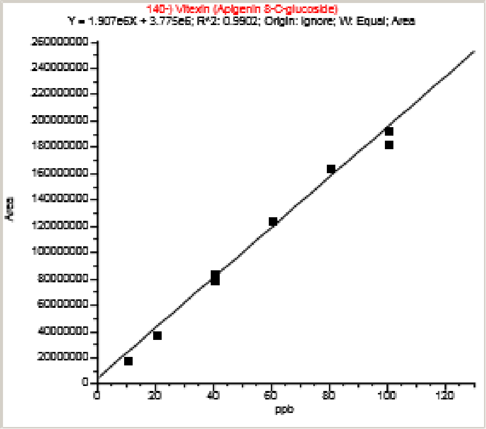

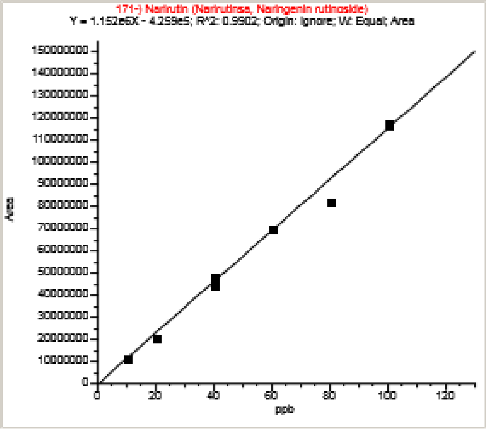

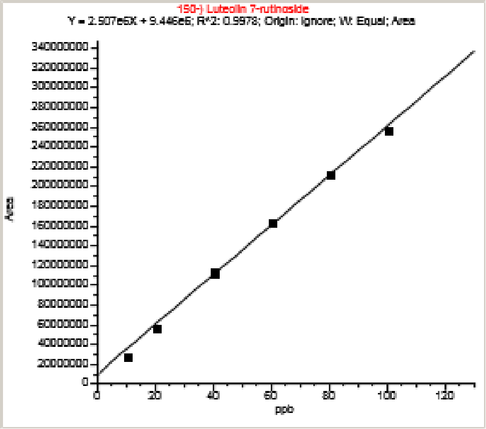

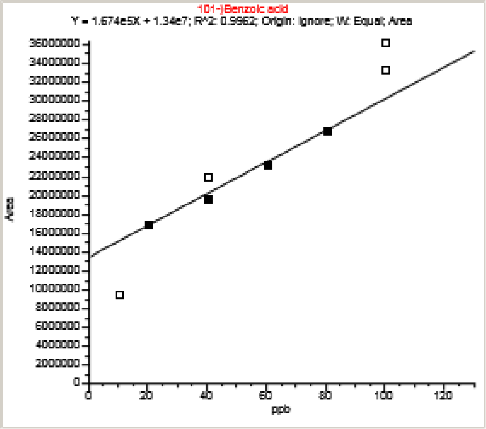

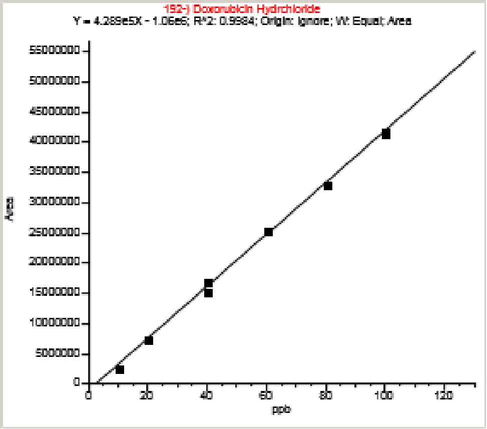

High Density Calibration Report

Lab Name: Default Laboratory
Instrument: Thermo Scientific Instrument
User: Thermo
Batch: FENOLICQUAN-13
Method: FENOLICQUAN-13_FENILICQUAN FENILICQUAN
Cali File: FENOLICQUAN-13.calx

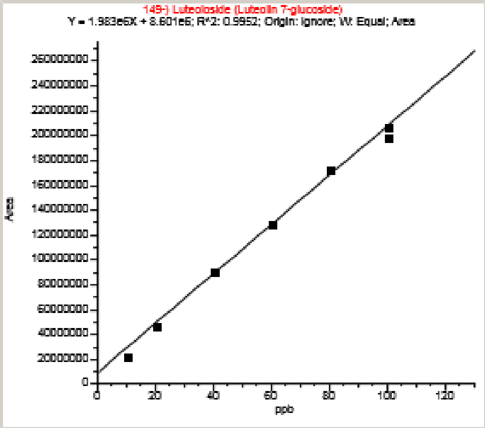

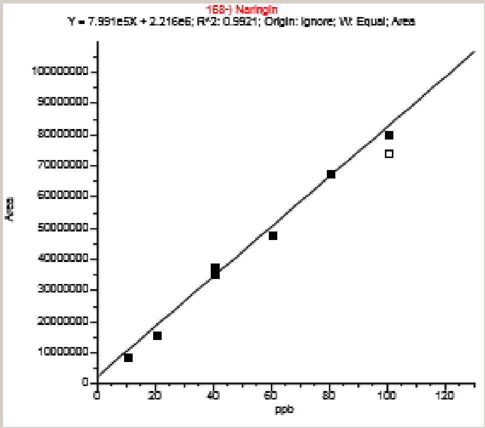

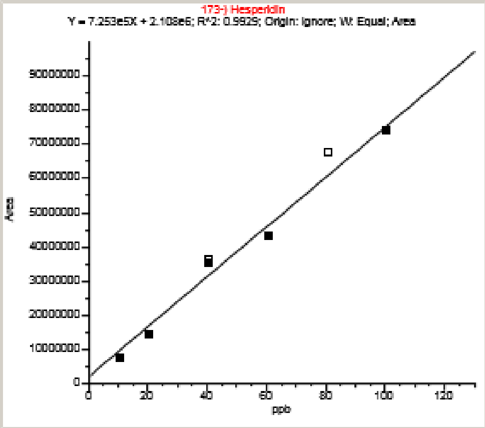

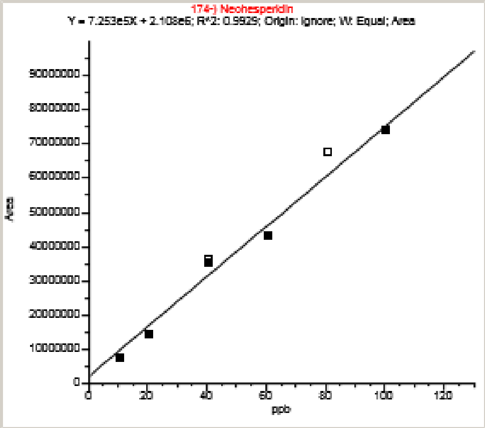

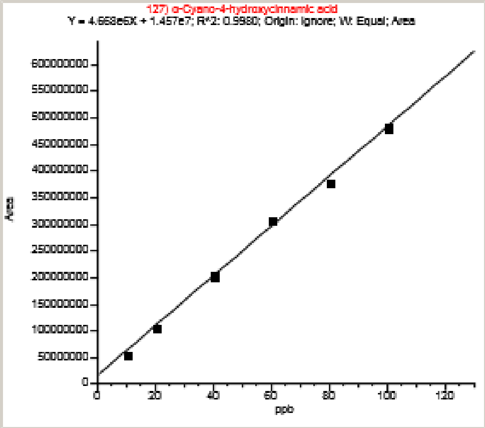

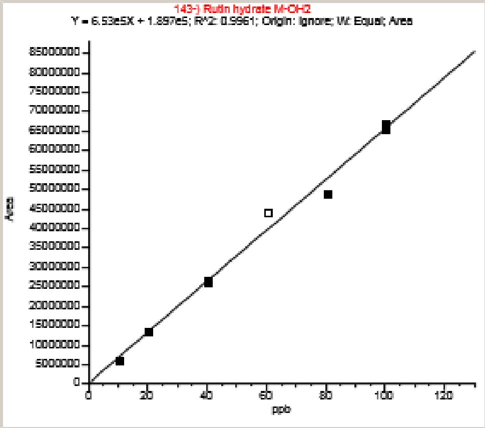

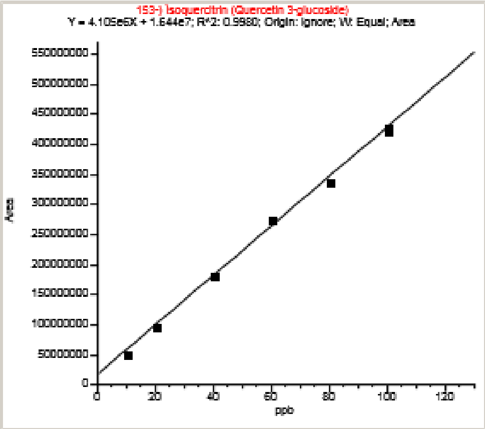

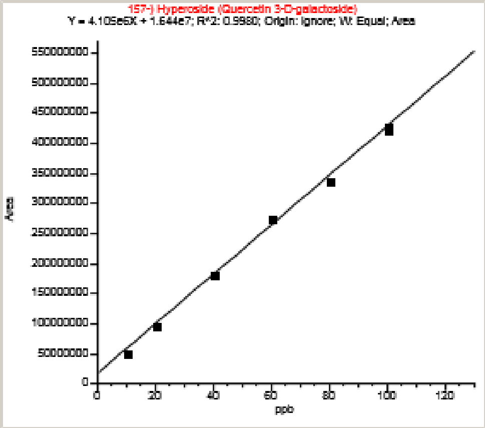

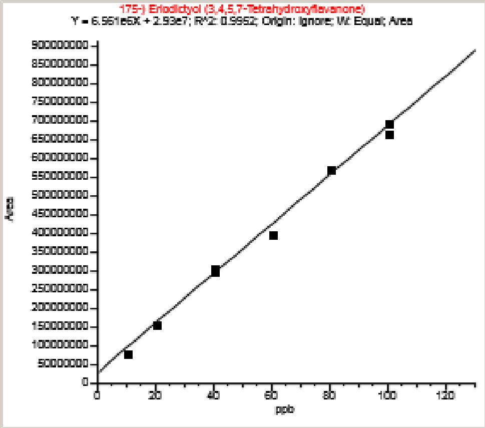

High Density Calibration Report

Lab Name: Default Laboratory
Instrument: Thermo Scientific Instrument
User: Thermo
Batch: FENOLICQUAN-13

Method: FENOLICQUAN-13_FENILICQUAN
FENILICQUAN
Cali File: FENOLICQUAN-13.calx

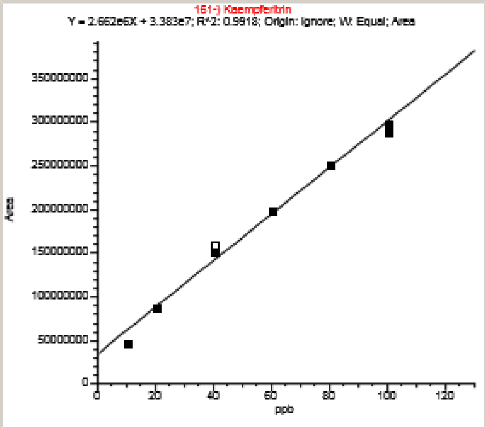

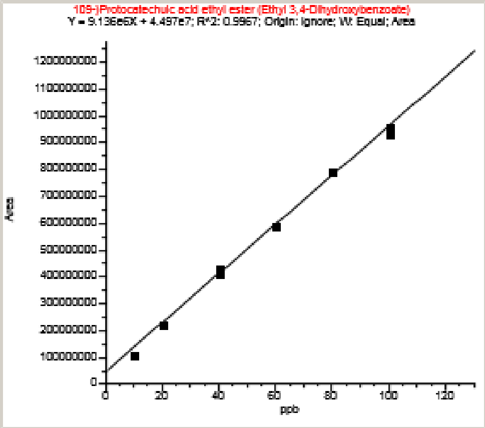

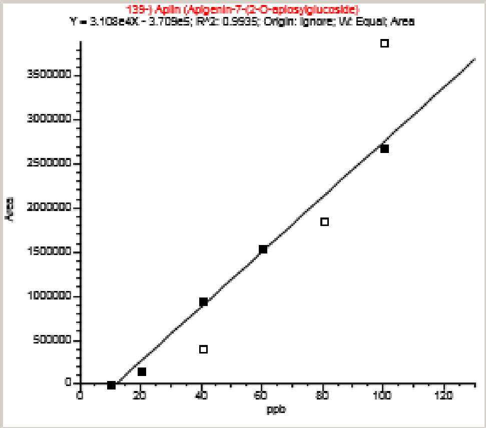

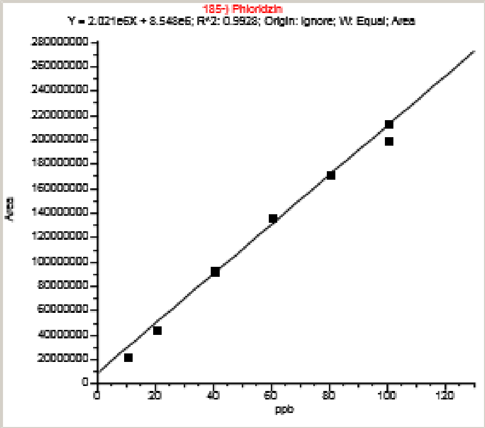

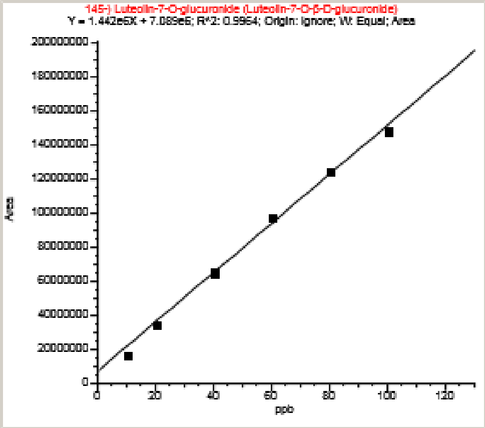

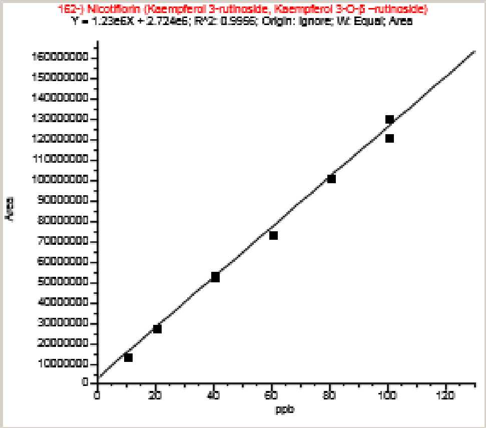

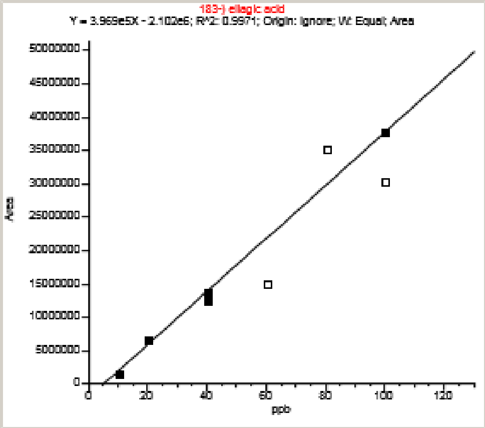

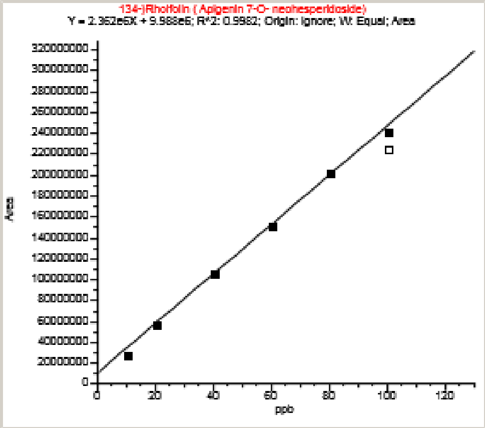

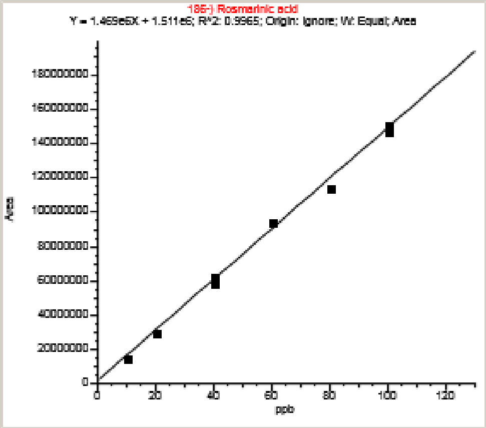

High Density Calibration Report

**Lab Name:**	Default Laboratory		
**Instrument:**	Thermo Scientific Instrument	**Method:**	FENOLICQUAN-13_FENILICQUAN
**User:**	Thermo		FENILICQUAN
**Batch:**	FENOLICQUAN-13	**Cali File:**	FENOLICQUAN-13.calx

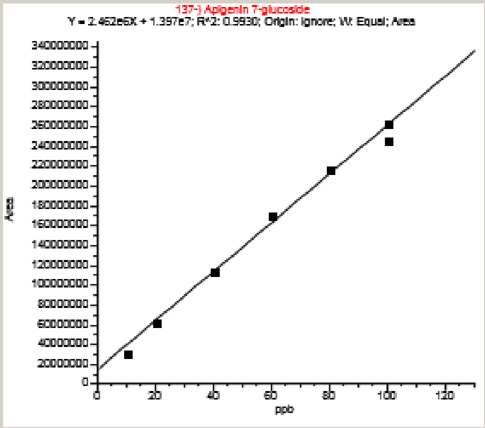

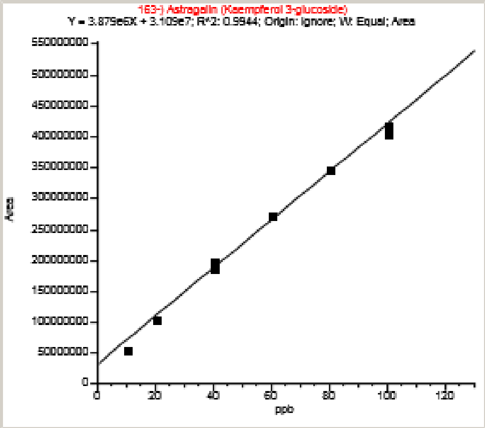

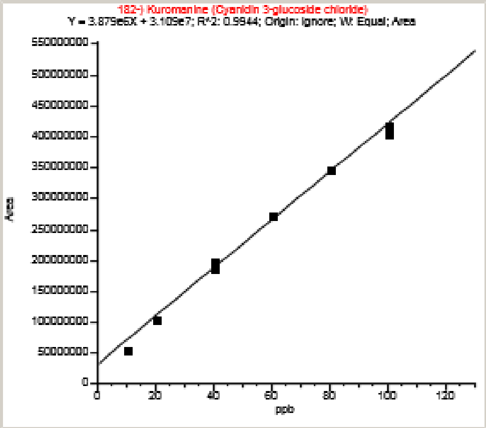

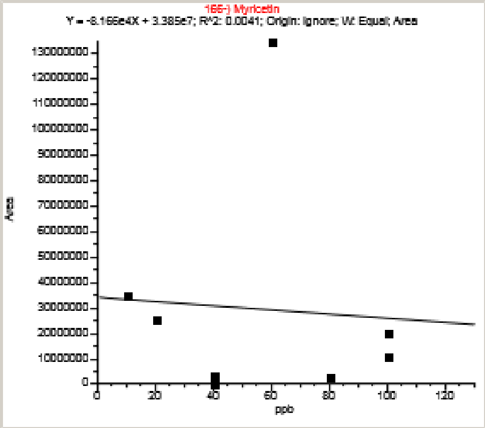

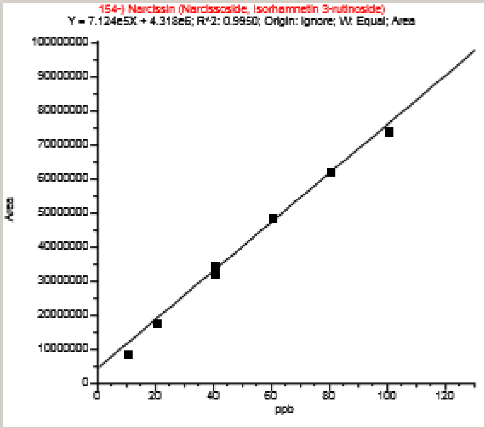

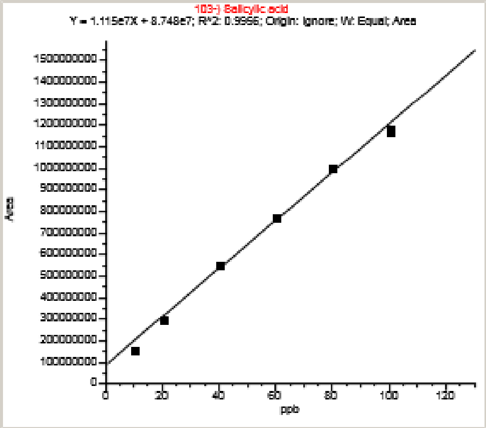

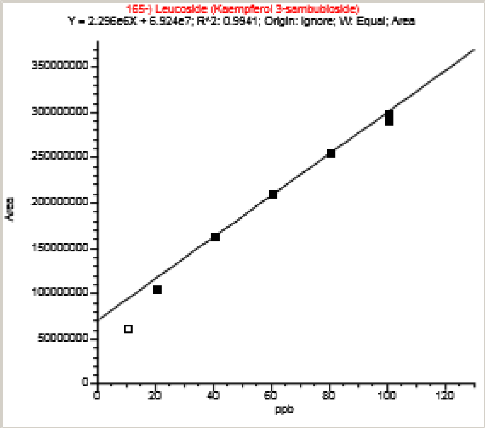

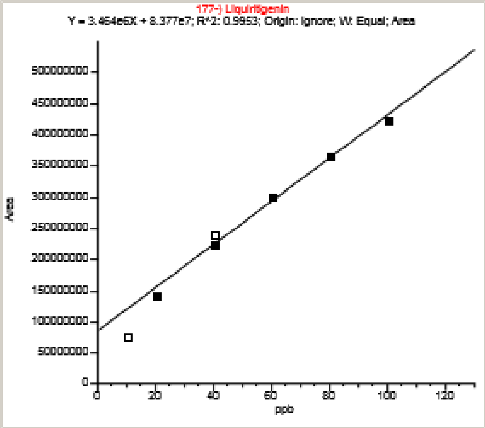

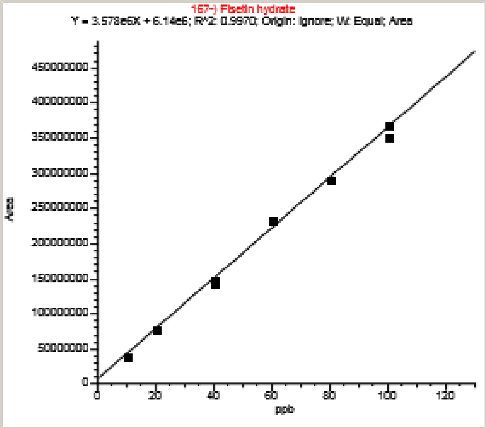

High Density Calibration Report

Lab Name: Default Laboratory
Instrument: Thermo Scientific Instrument
User: Thermo
Batch: FENOLICQUAN-13
Method: FENOLICQUAN-13_FENILICQUAN FENILICQUAN
Cali File: FENOLICQUAN-13.calx

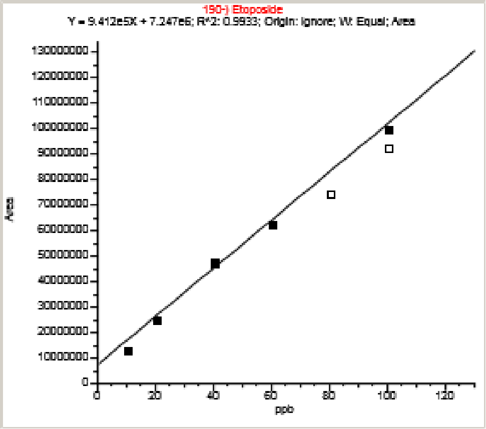

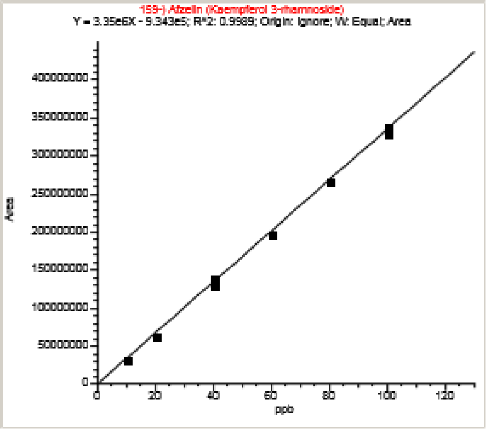

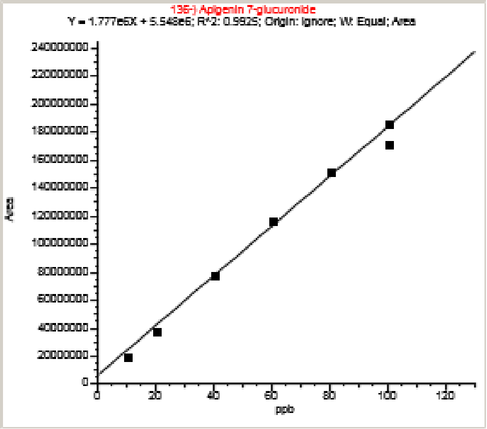

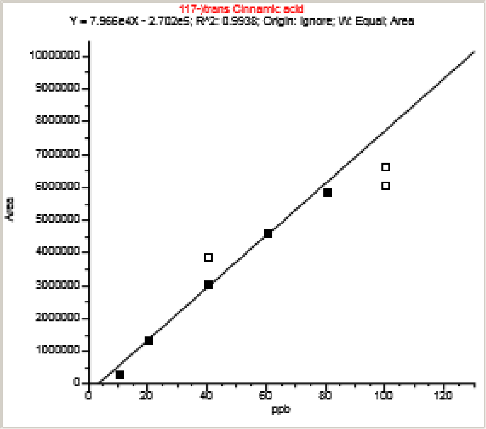

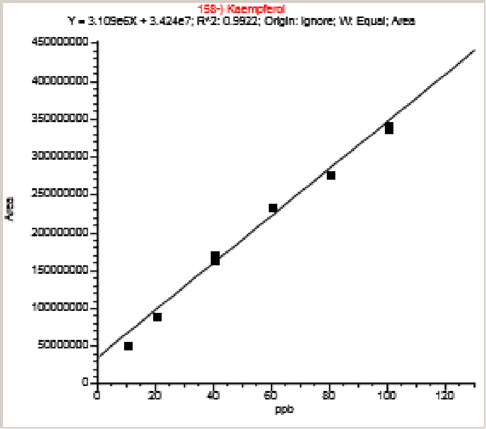

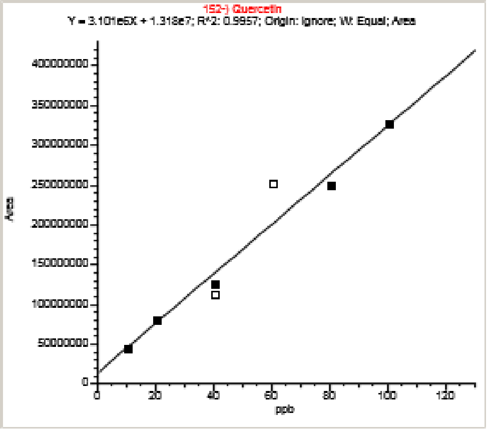

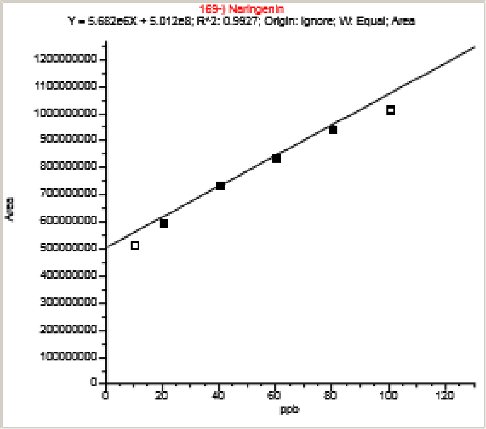

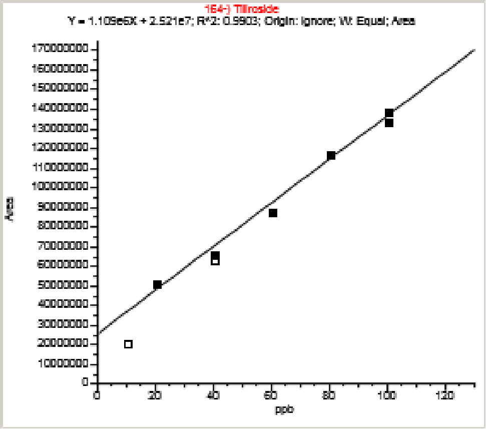

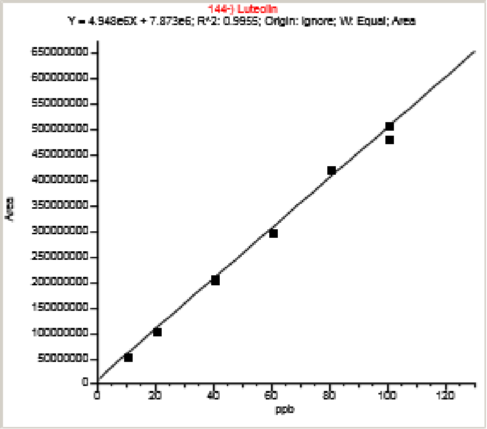

High Density Calibration Report

Lab Name: Default Laboratory
Instrument: Thermo Scientific Instrument
User: Thermo
Batch: FENOLICQUAN-13

Method: FENOLICQUAN-13_FENILICQUAN FENILICQUAN
Cali File: FENOLICQUAN-13.calx

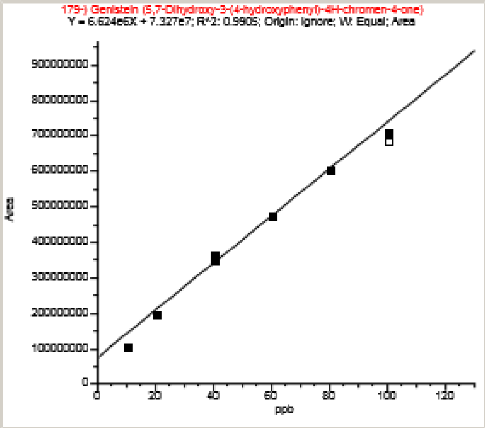

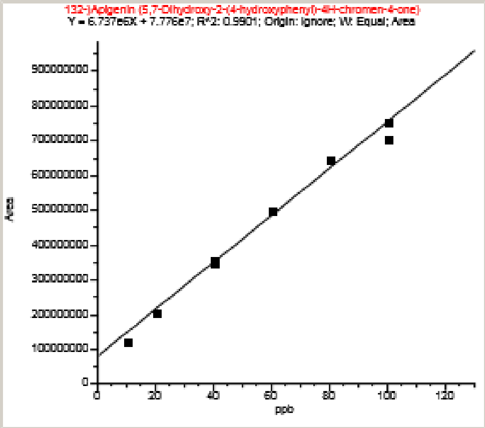

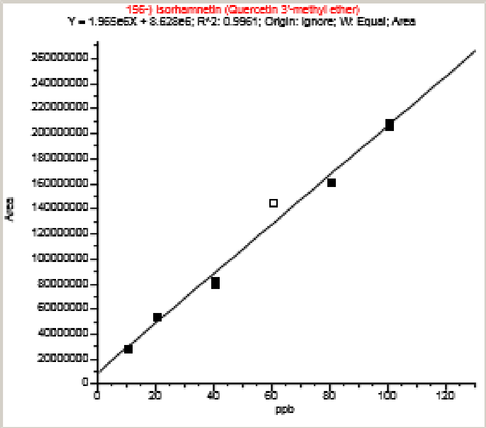

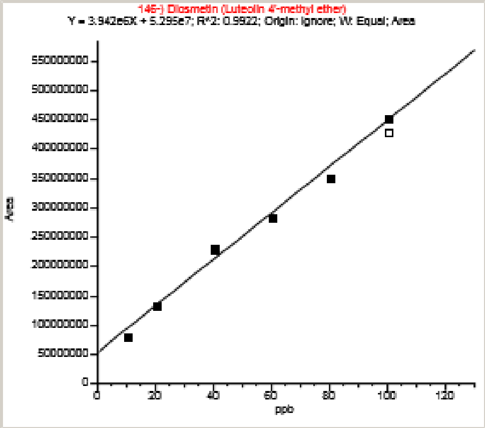

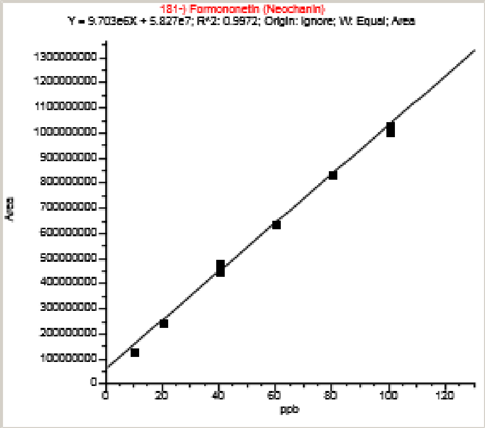

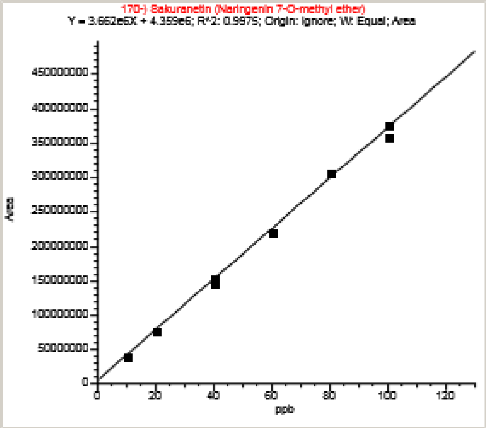

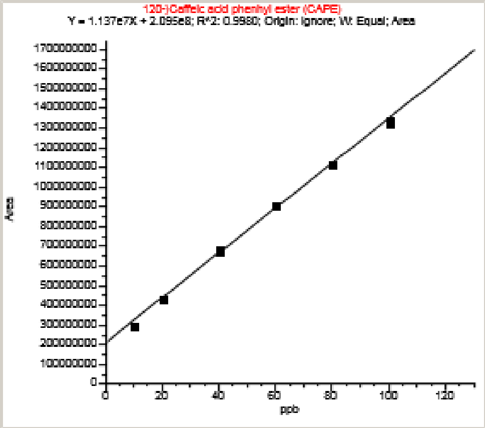

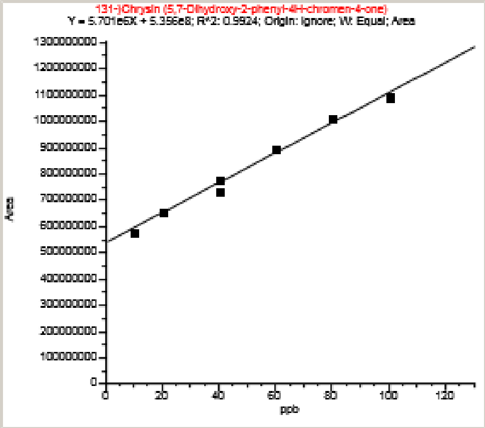

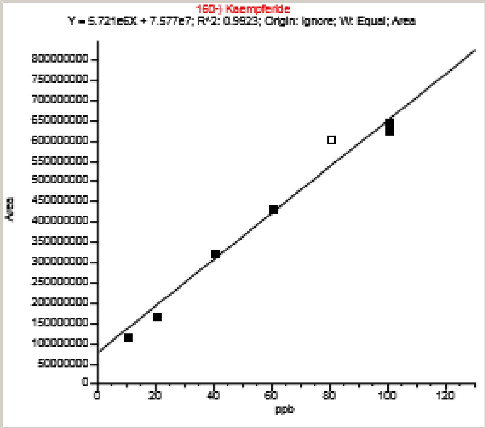

High Density Calibration Report

**Lab Name:**	Default Laboratory		
**Instrument:**	Thermo Scientific Instrument	**Method:**	FENOLICQUAN-13_FENILICQUAN
**User:**	Thermo		FENILICQUAN
**Batch:**	FENOLICQUAN-13	**Cali File:**	FENOLICQUAN-13.calx

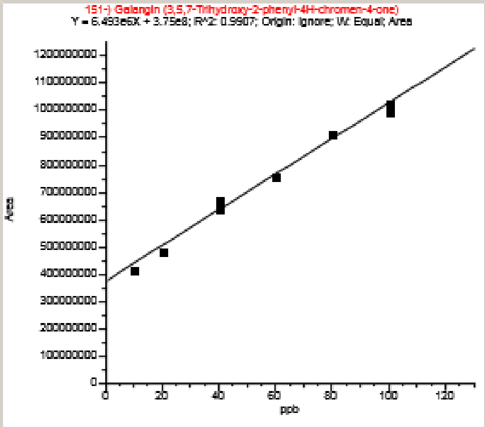

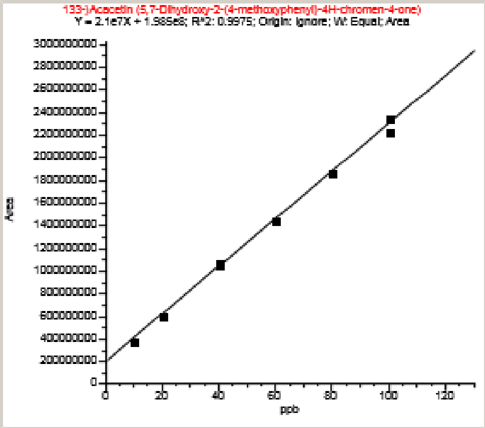

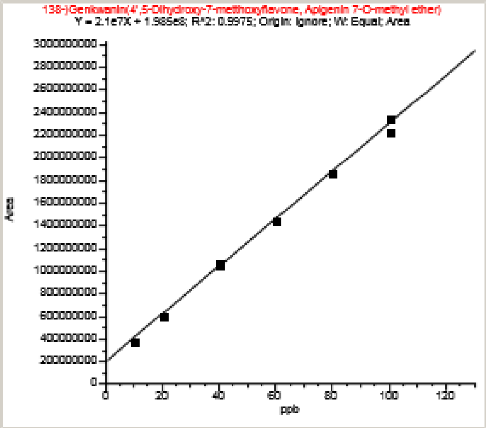

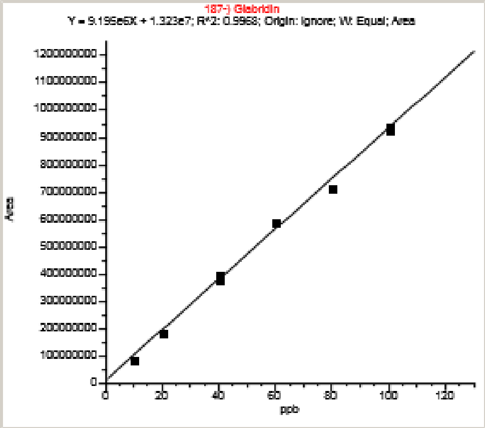

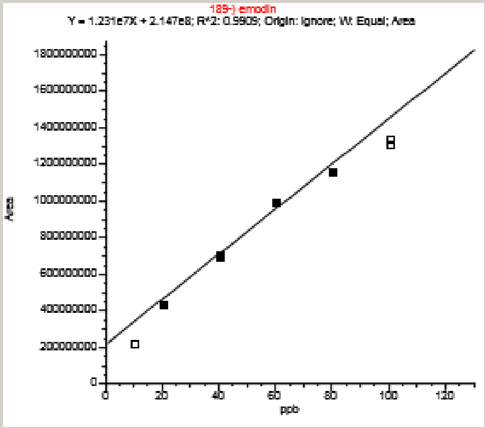

Supplement: Supplemental Information 3 [file peerj-12-18046-s003.pdf]
