# Supplementary material for: Phytochemical profiling and antioxidant activity assessment of Bellevalia pseudolongipes via liquid chromatography-high-resolution mass spectrometry
Source: PeerJ. 2024 Sep 13;12:e18046. doi: 10.7717/peerj.18046 (PMC11404456; doi:10.7717/peerj.18046)
Supplement: Supplemental Information 5 [file peerj-12-18046-s005.docx]

| **Rapor Numarası ve Tarihi** | *2023-1* |  |
| --- | --- | --- |
| **Numune Evrak Kayıt No** | 2023-1 |  |
| **Numune Adı/Cinsi** | bitki |  |
| **Numune Taşıma/Saklama Şartları** | Oda sıcaklığı |  |
| **Analizin Adı/Türü** | Toplam Fenolik, Toplam Flavonoid, DPPH, |  |
| **Kullanılan Metot Bilgisi** |  |  |
| **Analize Başlama Tarihi** | 02/01/2023 |  |
| **Analiz Süresi (saat/gün)** |  |  |
| **Analizden Sorumlu Personel** | Leyla ERCAN, Haşim ÇAVUŞOĞLU |  |
| **Fatura Edilecek Kişi/Kurum/Kuruluş** | Mardin Artuklu Üniversitesi Merkezi Araştırma Laboratuvarı Uygulama ve Araştırma Merkezi |  |
| **Makbuz Tarih ve No / Dekont Tarih ve No** |  |  |
|  | |  |
| **Açıklamalar:** 1) Numune tarafımızca alınmış olup, Merkezi Araştırma Laboratuvarı Numune Kabul prosedürüne uygun olarak kabul edilmiştir. 2) Bu rapordaki sonuçlar ve görüşler analizi yapılan numuneyi temsil eder ve başka amaçla kullanılamaz. Bu raporun hiçbir bölümü tek başına veya kısmen kullanılamaz ve Merkezi Araştırma Laboratuvarı’nın izni olmadan çoğaltılamaz. İmzasız raporlar geçersizdir. 3) Özel istek numune analiz raporları adli ve idari işlemlerde ve reklam amaçlı kullanılamaz. 4) Numune ile ilgili bilgiler müşteri tarafından sağlanmıştır. 5) Laboratuvarımızın personeli haricinde yürütülen numune alımlarında, analiz yapılan numunede; numune alımından laboratuvarımıza teslimine kadar olan prosedürlerin ve bakılması istenen grup ve parametrelerin belirlenmesinde teknik ve hukuki sorumluluk numuneyi alana aittir. Laboratuvara gelen numuneler metoduna uygun alınmış kabul edilir ve hatalı alınan numunelerden mesuliyet kabul edilmez. 6) Genişletilmiş ölçüm belirsizlikleri müşteri talebi veya yasal mevzuatlar zorunlu kıldığı durumlarda belirtilir. 7) Laboratuvar bu raporu yasal yükümlülükler dışında müşterinin izni olmadan üçüncü şahıslara gösteremez ve çoğaltamaz. Yasal otorite müşterinin haberi olmadan rapora ulaşmak isterse müşteriye bilgi verilmez. 8) Numune müşteri tarafından sağlandığı durumlarda, ölçüm belirsizliğinde numune almadan kaynaklanan katkı dâhil edilmemiş ve numune alındığı şekliyle analize tabi tutulmuştur. 9) Numune ile ilgili kişi ve kurumlar yukarıda belirtilen hususları kabul etmiş sayılır. | |  |
| **ANALİZ SONUÇLARI:**  **1-Toplam Fenolik Bileşik Tayin Sonuçları**  **Birim:** **sonuç mg gallik asit eşdeğeri/ g örnek**   \| **Numune** \| **Sonuç(±standart sapma)** \| \| --- \| --- \| \| A17 \| 0,24±0,004 \| \|  \|  \|   **2-Toplam Flavonoid Tayin Sonuçları**  **Birim:** **sonuç mg catechin eşdeğeri/ 100g örnek**   \| **Numune** \| **Sonuç(±standart sapma)** \| \| --- \| --- \| \| A17 \| 0,043±0,001 \| \|  \|  \|   **3- Antioksidan Kapasite Tayin Sonuçları (DPPH)**  **Birim:** **mg** **Troloks eşdeğeri /g örnek başına**   \| **Numune** \| **Sonuç** \| \| --- \| --- \| \| A17 \| 0,29±0,2 \| \|  \|  \|   **Referans Metodlar:**  **Toplam Fenolik Tayini:** Esra Çapanoğlu ve ark. Changes in polyphenol content during production of grape juice concentrate, Food Chemistry, 139,521-526,2013 Yöntemi modifiye edilerek  **Toplam Flavonoid Tayini**: Zhishen, J., Mengcheng, T., ve Jianming, W. (1999) . The Determination of Flavonoid contents in mulberry and their scavening effects on superoxide radicals. Food Chemistry, 64,555-559 Yöntemi modifiye edilerek  **DPPH Antioksidan Tayini:**Makhlouf ve ark., Ultrafiltration and thermal processing effects on Maillard reaction products and biological properties of date palm sap syrups(Phoenix dactylifera L.), Food Chemistry,256,397-404 Yöntemi modifiye edilerek   \| Kimyager Leyla ERCAN \|  \| Haşim ÇAVUŞOĞLU \| \| \| --- \| --- \| --- \| --- \| \|  \|  \| \|  \| \| | |  |

**Laboratuvar Müdürü**

**Dr. Öğretim Üyesi Muhammed GÜNGÖREN**
